# AI-Based Approaches to Refugee Mental Health Care: Systematic Integrative Review

**DOI:** 10.2196/101857

**Published:** 2026-08-26

**Authors:** Hun Kang, Subin Park, Jooyoung Kim, Ocksim Kim, Hokon Kim, JiYeon Choi, Sang Hui Chu

**Affiliations:** 1 College of Nursing Yonsei University Seoul, Seoul Republic of Korea; 2 Brain Korea 21 FOUR Project College of Nursing Yonsei University Seoul, Seoul Republic of Korea; 3 Department of Nursing Dongnam Health University Suwon, Gyeonggi-do Republic of Korea; 4 Institute for Innovation in Digital Healthcare Yonsei University Seoul, Seoul Republic of Korea; 5 Mo-Im Kim Nursing Research Institute College of Nursing Yonsei University Seoul, Seoul Republic of Korea; 6 Institute of Population and Human Capital Yonsei University Seoul Republic of Korea

**Keywords:** artificial intelligence, mental disorders, mental health, refugees, review

## Abstract

**Background:**

Refugees and forcibly displaced populations experience elevated rates of mental health conditions, including posttraumatic stress disorder, depression, and anxiety, while facing substantial barriers to mental health care. AI has emerged as a promising approach for mental health detection, intervention, and decision support; however, no review has specifically examined AI-based approaches to refugee mental health care.

**Objective:**

This systematic integrative review aimed to (1) examine how AI has been used in refugee mental health research and care, (2) synthesize the reported strengths and limitations of AI-based approaches, and (3) identify future directions for this field.

**Methods:**

An integrative review methodology was used following Whittemore and Knafl’s framework. Systematic searches were conducted across PubMed, Embase, CINAHL, PsycINFO, Scopus, Web of Science, Cochrane Library, ACM Guide to Computing Literature, IEEE Xplore, arXiv, and medRxiv from inception through June 2026. The first 100 results from Google Scholar sorted by relevance were additionally screened. Studies were included if they focused on refugee populations and applied AI technologies to refugee mental health care or assessment, while studies focusing on voluntary migrants and digital mental health not using AI were excluded. Quality appraisal was performed mainly using the Mixed Methods Appraisal Tool. Data were synthesized in accordance with the integrative review methodology.

**Results:**

Of 2726 studies identified, 23 studies were included in the review. AI-based approaches were classified into 3 functional categories: Detection, Prediction, and Classification (15/23, 65.2%); Intervention and Clinical Support (4/23, 17.4%); and Information Synthesis (5/23, 21.7%). Reported strengths of AI-based approaches included scalability, accessibility in resource-limited settings, support for early symptom detection, reduction of language barriers, and efficient synthesis of large unstructured data. Common limitations included potential biases, lack of contextual understanding, structural barriers in technology access, and concerns regarding overreliance on AI-based systems. Future directions emphasized culturally sensitive model development, larger, multimodal datasets, clinical validation, human-centered design, and implementation research.

**Conclusions:**

Unlike previous reviews that focused primarily on digital mental health interventions or AI applications in general populations, this review provides the first comprehensive synthesis of AI-based approaches specifically for refugee mental health care. This review identified 3 functional domains that characterize the current landscape of AI applications in refugee mental health care and highlights key methodological, ethical, and implementation considerations. Despite heterogeneity in the included studies, the findings provide a foundation for future research and may inform the responsible development, evaluation, and implementation of AI-based approaches in refugee mental health care across humanitarian settings.

**Trial Registration:**

PROSPERO CRD420251246982; https://www.crd.york.ac.uk/PROSPERO/view/CRD420251246982

## Introduction

Globally, the number of forcibly displaced individuals is at the highest level, exceeding 117 million by the end of June 2025 [1]. This population encompasses refugees, internally displaced people, asylum seekers, and those in need of international protection due to persecution, conflict, and violence [1]. Furthermore, the escalating climate crisis has forced a growing number of people to leave their homes, leading to the emergence of climate change refugees [2]. While these individuals differ in their legal status and whether they are eligible for international protection under international law [3-5], they—hereafter referred to collectively as refugees—are frequently exposed to various stressors and traumatic events before, during, and after displacement. Such experiences contribute to a high prevalence of mental health conditions, including posttraumatic stress disorder (PTSD), depression, and anxiety [6,7]. However, mental health services in humanitarian contexts are severely underresourced, hampered by a shortage of trained health professionals, language barriers, and fragmented infrastructure [8,9]. Consequently, there is an urgent need for innovative and scalable approaches that address these structural limitations of traditional mental health systems.

AI has advanced rapidly and shown significant promise in mental health care, including early detection and diagnosis, intervention and treatment, and symptom monitoring [10-13]. AI, defined as computer systems capable of performing complex tasks that typically require human intelligence, encompasses various technologies that can be incorporated in health care, such as machine learning, large language models (LLMs), and natural language processing (eg, sentiment analysis and speech recognition) [12,13]. For example, machine learning models have leveraged multimodal data—including physiological measures, audio and speech signals, and questionnaires—to detect and predict stress-related mental disorders [14], while AI-based conversational agents have been tested and used to promote mental health and well-being [15,16]. These technologies are particularly well-suited for refugee populations. For example, machine learning models have been used to predict PTSD symptoms among Ukrainian refugees using questionnaire data [17], and to detect psychological trauma in refugee children by analyzing drawings [18]. Additionally, a speech-to-speech translation system that safeguards patient data privacy has been developed to overcome linguistic barriers for refugees in psychiatric treatment [19].

While recent reviews have synthesized the literature on AI-based approaches to mental health care [10,11,20], no review has focused specifically on refugees. In contrast, several reviews have examined digital mental health interventions and applications for refugees [21,22]. However, these reviews did not specifically evaluate AI-based applications, while AI extends beyond digital technologies, encompassing functions such as predictive modeling, synthesis of large volumes of data, and generation of new content [23-25]. In addition, AI raises unique methodological and ethical concerns; for example, most AI models are trained on data derived from Western, educated, industrialized, rich, and democratic populations [26,27], potentially creating significant algorithmic bias when generalized to non-Western populations. As such, refugee population presents unique challenges for AI deployment, including heightened psychiatric vulnerability, linguistic barriers and cultural differences in host countries, and profound concerns regarding privacy, security, and surveillance [9,28,29]. Given these complexities, a comprehensive review specific to refugee populations is necessary to map existing applications, identify methodological and ethical considerations, and evaluate the potential for AI to enhance refugee mental health support. Such a review is particularly timely, given the rapid advancement of AI technologies alongside the unprecedented number of refugees and forcibly displaced people worldwide [1].

To address this gap, this systematic integrative review aimed to (1) examine how AI has been used in refugee mental health research, (2) document the strengths and limitations of these AI-based approaches, and (3) explore future directions for this field. An integrative review methodology was selected to accommodate the diverse range of study designs spanning both the health sciences and computing disciplines. Findings will guide future research, inform policy and clinical practice, and support culturally sensitive integration of AI into refugee mental health care.

## Methods

### Study Design

This review used an integrative review methodology proposed by Whittemore and Knafl [30]. An integrative review is considered the broadest type of review methodology, encompassing diverse study designs and data types and serving a wide range of purposes including defining concepts, reviewing theories and evidence, and analyzing methodological issues of a certain topic [30]. An integrative review was considered the most appropriate approach for this study, given (1) the interdisciplinary and methodologically diverse nature of the topic—which is reflected in the included studies as well—and (2) the study aims to review existing evidence and identify and discuss methodological strengths, limitations, and future directions in AI-based approaches to refugee mental health care. Additionally, considering the aims of this review, meta-analysis was deemed not suitable and therefore not conducted.

The protocol of the review was preregistered with PROSPERO (International Prospective Register of Systematic Reviews; CRD420251246982). There was one deviation from the registered protocol: while we did not restrict the search date in the protocol, we limited our search to studies published up to June 18, 2026—the date of the second round of literature search conducted to identify up-to-date studies. This review was conducted in accordance with the PRISMA (Preferred Reporting Items for Systematic Reviews and Meta-Analyses) guidelines [31]. Because this review did not include a quantitative synthesis (eg, meta-analysis), PRISMA items related to quantitative synthesis were marked as not applicable. The completed PRISMA 2020 checklist and PRISMA 2020 for Abstracts checklist are provided in Multimedia Appendices 1 and 2, respectively.

### Information Sources and Search Strategy

The PRISMA-S (Preferred Reporting Items for Systematic Reviews and Meta-Analyses literature search extension) checklist is provided in Multimedia Appendix 3 [32]. To identify relevant studies, we conducted systematic literature searches in the following electronic databases from their respective inceptions through December 2025: PubMed, Embase, CINAHL, PsycINFO, Scopus, Web of Science, Cochrane Library, the ACM Guide to Computing Literature, and IEEE Xplore. Given the rapid evolution of the AI field, we also searched arXiv and medRxiv to identify relevant unpublished manuscripts and preprints. Each database was searched individually rather than through a single search platform. We conducted an updated search in June 2026 to identify newly published studies. As a supplementary method to maximize search comprehensiveness, we additionally screened the first 100 records sorted by relevance in Google Scholar, informed by a previous scoping review [20]. Additionally, we conducted backward and forward citation searching of the included studies. Potentially relevant studies were identified by examining the reference lists of the included studies (backward citation searching) and examining the studies citing the included studies on Google Scholar (forward citation searching). No study registries were searched, and no additional studies or data were sought by contacting authors, experts, or other individuals. No restrictions were applied during the database searches, and no published search filters were used. Search strategies were developed by the research team, incorporating keywords and MeSH terms informed by previous literature, and were subsequently reviewed by a medical librarian prior to conducting the database searches. The search strategies for each database are provided in Multimedia Appendix 4.

### Eligibility Criteria

Studies were included if they (1) focused on refugee populations, including asylum seekers, forcibly displaced persons, and humanitarian migrants; and (2) explored the application of AI (eg, machine learning and natural language processing, LLMs) in any aspect of mental health assessment and care. We adopted a broad operational definition of refugees, including individuals who fled their homes to other regions in their home country (ie, internally displaced persons) or to other countries (ie, refugees) to seek safety, regardless of whether they had been granted international protection (ie, refugees) or were awaiting such protection (ie, asylum seekers) [3-5]. We also included individuals who were displaced to other regions or countries to flee from climate-related disasters (ie, climate change refugees) [2].

Exclusion criteria were (1) studies focusing on immigrants or migrants who voluntarily relocated, (2) research on digital health or technology-based health care that did not use AI, (3) unpublished theses and dissertations, editorials, reviews, book chapters, conference poster abstracts, and clinical registrations, and (4) studies published in languages other than English. We adopted strict eligibility criteria for the target population, thereby excluding migrants who voluntarily relocated, considering the traumatic experiences that refugees undergo before, during, and after migration and the impact of these experiences on mental health [33,34]. If studies did not specify the target population as refugees as indicated above, they were excluded; however, if refugees and immigrants were examined together, such studies were included in the review.

### Selection Process

Following the systematic search, identified records were exported to Covidence (Veritas Health Innovation Ltd), a specialized review management platform, which automatically removed duplicate records. Title and abstract screening was performed by pairs of independent reviewers, with any disagreements resolved by a third reviewer. Subsequently, pairs of independent reviewers conducted full-text screening, with discrepancies resolved through discussion within the research team.

### Quality Appraisal of the Selected Studies

The quality of included studies was mainly appraised using the Mixed Methods Appraisal Tool (MMAT), which was designed to evaluate qualitative, quantitative randomized controlled trials, quantitative nonrandomized studies, quantitative descriptive studies, and mixed methods study designs [35]. Quality appraisal was initially performed by the first author and verified by another author. Given the heterogeneity of study designs of the included studies, methodological quality was assessed using the MMAT only for empirical health research studies. The theoretical paper on the ethics of AI use was appraised using the Joanna Briggs Institute (JBI) Critical Appraisal Checklist for Text and Opinion Papers [36]. Studies primarily focused on the development, optimization, or technical evaluation of AI systems were not formally appraised because no widely accepted appraisal tool currently exists for the evaluation of engineering- and computing-focused studies.

### Data Collection Process

The first author extracted data, and another author verified it. Extracted data included study characteristics (author, publication year, peer-review status, and study aim), refugee characteristics (refugee background and country of residence at data collection), AI-related characteristics (purpose of AI use, AI technology used, and input data), mental health conditions assessed, and the reported strengths, limitations, and future directions of AI-based approaches to refugee mental health care.

### Synthesis Methods

Data were synthesized in accordance with the integrative review methodology, which involves data reduction, data display, data comparison, and conclusion drawing and verification [30]. For data reduction and display, key characteristics and findings were extracted and organized into summary tables. Specifically, information regarding the purpose of AI use was analyzed using an inductive thematic approach. The inductive approach was chosen given that AI-based approaches to refugee mental health represent an emerging field and thus lack an established theoretical framework. Initially, the primary purpose of AI use in each study was coded into concise keywords. These keywords were then iteratively compared based on their conceptual similarities, which allowed three overarching functional categories of AI to emerge inductively.

For data comparison, these categories were examined independently to identify patterns, similarities, and differences across studies, with regard to AI use, types of AI technology, and input data within each category. Additionally, strengths, limitations, and future directions of AI use in this setting were examined and compared to identify themes within each functional category.

Finally, during conclusion drawing and verification, an iterative review process was used to derive and verify insights. Specifically, an integrated summary table of the strengths, limitations, and future directions of AI-based approaches to refugee mental health was generated to provide a comprehensive portrayal of the findings. The synthesis was led by the first author and discussed and verified within the research team to generate the final insights.

## Results

### Study Selection

**Figure 1:**
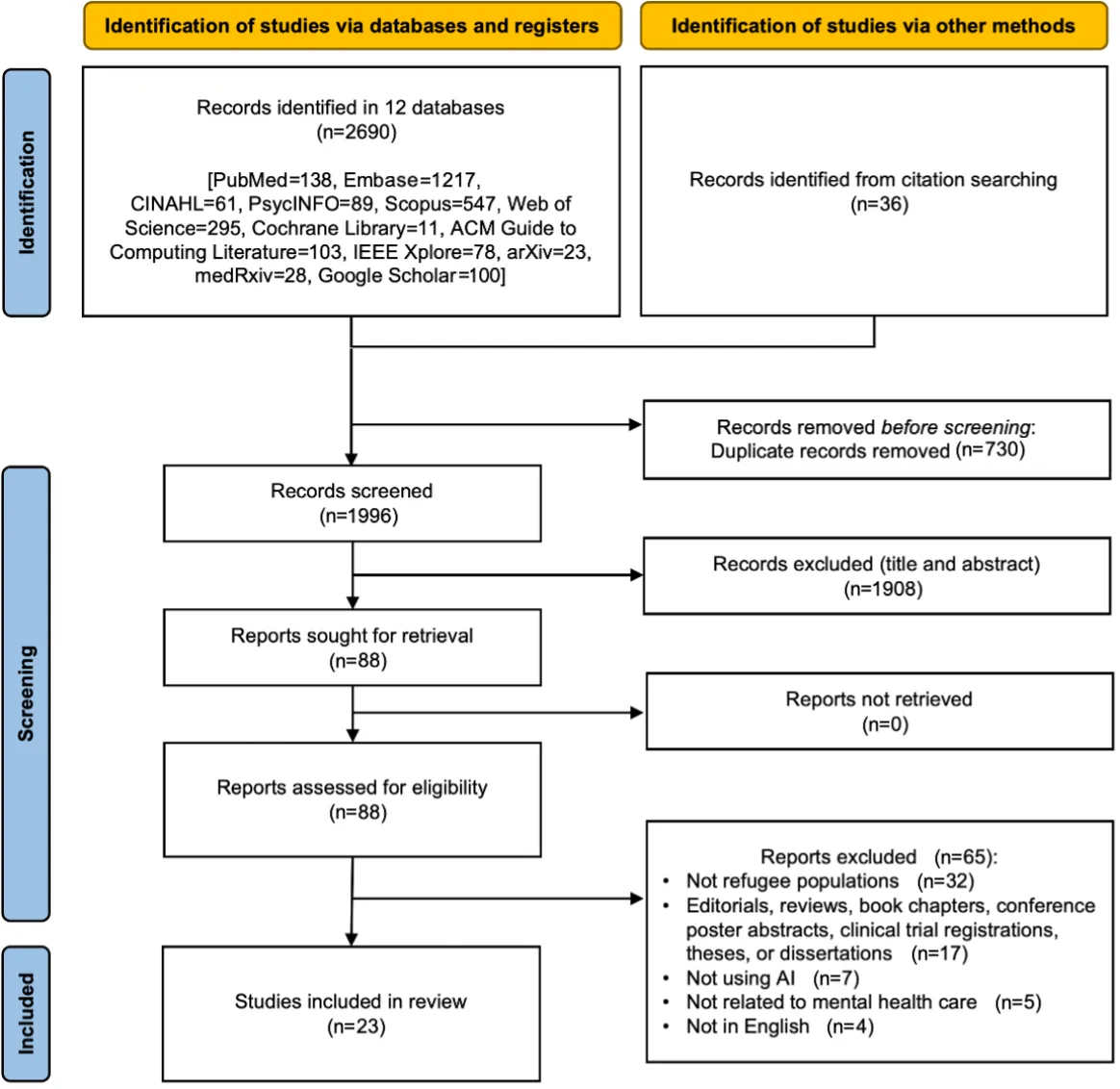
PRISMA (Preferred Reporting Items for Systematic Reviews and Meta-Analyses) flowchart of literature search and selection process.

Figure 1 summarizes the literature search and study selection process. Across 12 databases, we identified 2690 records: 138 from PubMed, 1217 from Embase, 61 from CINAHL, 89 from PsycINFO, 547 from Scopus, 295 from Web of Science, 11 from Cochrane Library, 103 from the ACM Guide to Computing Literature, 78 from IEEE Xplore, 23 from arXiv, 28 from medRxiv, and 100 from Google Scholar. Additionally, 36 records were identified from citation searching. After excluding 730 duplicates, we conducted a title and abstract screening for 1996 records, where 1908 records were excluded. Subsequently, 88 full-text articles were assessed for eligibility, and 65 articles were excluded. As a result, 23 studies were included for the review.

### Study Characteristics

Table 1 summarizes the characteristics of the 23 included studies. Most studies were peer-reviewed (22/23, 95.7%), except for 1 study. There was an increasing trend in the number of publications, with 1 publication in 2017, 2 in 2021, 3 in 2022, 2 in 2023, 5 in 2024, and 7 in 2025 (and an additional preprint). A total of 8 studies specifically targeted children and adolescent refugees, including one assessing parent-adolescent dyads (8/23, 34.8%), while the remaining targeted adults or did not specify.

**Table 1:** Key characteristics of the included studies.

Author (year)	Country of residence/refugee background	Mental health conditions of interest	Study aim
Augsburger (2017) [37]	Germany/displaced individuals from various countries	PTSD^a^ and depressive symptoms	To examine whether trauma exposure types predicted risk behaviors among displaced individuals who resettled in Germany
Mühl (2021) [38]	Germany/underaged refugees	PTSD, depression	To develop and evaluate an AI-based smartphone tool for PTSD screening in underage refugees using speech emotion recognition
Khatua (2021) [23]	Not specified/migrants and refugees on Twitter	Sufferings and mental stresses due to traumatic experiences	To analyze migrants’ and refugees’ concerns using social media data
Bhattacharya (2022) [18]	Bangladesh, India/Rohingya refugee children	PTSD	To develop a machine learning framework using children’s drawings to identify psychological trauma
Baird (2022) [39]	Jordan/Syrian refugee children	Psychological distress and trauma, PTSD, anxiety, depression	To examine associations between children’s drawing features, psychological outcomes, violence exposure, and host-community reintegration
Qasrawi (2022) [40]	West Bank/students in fifth to ninth grades in public schools and UNRWA^b^ schools	Depression, anxiety	To evaluate the accuracy of five machine learning models in predicting depression and anxiety, and related risk factors among Palestinian school children
Ugan (2023) [19]	Germany/not specified	Not specified	To develop a privacy-focused modular speech-to-speech translation system
Kamińska (2023) [41]	Poland/refugees (mainly Ukrainian refugees)	Stress	To evaluate the feasibility of classifying refugee stress levels using EEG^c^/GSR^d^ data
Sahoo (2024) [24]	India/Sri Lankan refugees at Trichy camp	Depressive symptoms, hostility, anxiety symptoms, somatization	To identify refugee mental health stressors and develop a predictive model for mental health
Pozzi (2024) [42]	Lebanon/Syrian refugees	Not specified	To discuss the ethical and epistemological implications of mental health chatbots for vulnerable populations
Dell (2024) [43]	United States/adult survivors of Hurricane Maria who migrated from Puerto Rico	Depression, PTSD, and anxiety symptoms	To assess the contribution of migration-related cultural stress features and compare random forest with logistic regression in classifying probable PTSD and depression
So (2024) [44]	South Korea/North Korean defectors	PTSD, complex PTSD, depressive disorder, anxiety disorder, alcohol use disorder	To evaluate whether LLMs can identify psychiatric symptoms and summarize stressors and symptoms from interview transcripts
Saleh (2024) [45]	India/parent-adolescent dyads from the Trichy refugee camp	Depressive symptoms	To examine the effects of parental transmigration stress on child depressive symptoms in displaced families
Shrivastava (2025^e^) [46]	Not specified/not specified	Not specified	To develop and evaluate a RAG^f^-based AI framework for extracting refugee mental health data while reducing LLM^g^ hallucinations
Ashqar (2025) [47]	Lebanon/Palestinian refugees who experienced Nakba in 1948	Not specified	To investigate whether LLMs can accurately analyze emotional tone in Palestinian refugee testimonies and assess the impact of summarization on sentiment representation
Bashar (2025) [48]	Bangladesh/adult Rohingya refugees	PTSD, depression, anxiety, insomnia	To introduce an interpretable data-driven approach for diagnosing PTSD, depression, anxiety, and insomnia among Rohingya refugees
Khatua (2025) [25]	Not specified/migrants and refugees on Twitter	Distress, depression, anxiety	To summarize unstructured social media data for use in generative agent-based simulations to inform policy interventions
Habashneh (2025) [49]	Jordan/Syrian adolescent refugees	Depression	To predict depressive symptoms among Syrian adolescent refugees
Figueiredo (2025) [17]	Portugal/adult Ukrainian refugees	PTSD	To predict PTSD risk among adult Ukrainian refugees using machine learning algorithms
Keller (2025) [50]	Germany/unaccompanied young refugees	Suicidal ideation, PTSS^h^, depression	To examine suicidal ideation and associated risk and protective factors among unaccompanied young refugees
Gellert (2025) [51]	Poland/Ukrainian (including refugees and displaced persons) and Polish patients	Anxiety, depressed moods, GAD^i^, fear, irritability, insomnia, nervousness, stress-related gastric symptoms, suicidal thoughts and intents	To assess mental health symptom reporting among Polish and Ukrainian patients using an AI-based virtual triage platform in the first year of the Russia-Ukraine War
Im (2026) [52]	South Sudan/refugees, internally displaced persons, and returnees from Sudan, Democratic Republic of the Congo, Central African Republic, Ethiopia, South Sudan, and others	Depressive symptoms	To examine health, socioeconomic, protection, and contextual factors associated with depressive symptom severity and quantify their relative contributions among adults living in displacement-affected settings
Demetry (2026) [53]	Sweden, Denmark, Germany/Arabic-speaking refugees or migrant adults living in Sweden, Denmark, and Germany	Not specified	To compare the cultural relevance and acceptability of CBT^j^ techniques adapted by AI vs human psychologists

^a^PTSD: posttraumatic stress disorder.

^b^UNRWA: United Nations Relief and Works Agency for Palestine Refugees.

^c^EEG: electroencephalogram.

^d^GSR: galvanic skin response.

^e^Submission year was used in place of publication year for a non–peer-reviewed study.

^f^RAG: retrieval-augmented generation.

^g^LLM: large language model.

^h^PTSS: posttraumatic stress symptoms.

^i^GAD: generalized anxiety disorder.

^j^CBT: cognitive behavioral therapy.

### Results of Quality Appraisal

Of the included studies, quality appraisal was conducted for 12 studies. Studies primarily focused on the development, optimization, or technical evaluation of AI systems were not formally appraised given the absence of a widely accepted appraisal tool for these studies. Among 11 studies assessed using the MMAT, 3 (27.3%) studies met all criteria, 6 (54.5%) studies met 80% of the criteria, 1 (9.1%) study met 60% of the criteria, and 1 (9.1%) study met 40% of the criteria. One study assessed using the JBI Critical Appraisal Checklist for Text and Opinion Papers met all 6 criteria. Multimedia Appendix 5 [17,24,35-37,39,40,42,43,45,50-53] provides results of quality appraisal for each study. Given the mapping purpose of this systematic integrative review, the quality appraisal was intended to contextualize the empirical health research rather than to weight individual studies during synthesis.

### Functional Categories of AI-Based Approaches to Refugee Mental Health Care

Based on the primary purpose of AI use, studies were inductively classified into 3 overarching functional categories, as summarized in Table 2.

**Table 2:** Functional categories of AI-based approaches to refugee mental health care.

Author (year)			Specific purpose of AI use		AI technology		Input data
**Category 1: Detection, Prediction, and Classification**							
	Augsburger (2017) [37]	Prediction of risk-taking behaviors (depression and PTSD^a^ symptoms as predictors)		Machine learning		Survey-based data, behavioral task	
	Mühl (2021) [38]	Detection of PTSD		Machine learning, speech-emotion recognition		Survey-based data, audio data	
	Bhattacharya (2022) [18]	Detection of psychological trauma		Machine learning		Drawings	
	Baird (2022) [39]	Prediction of psychological distress		Machine learning		Drawings, survey-based data, administrative refugee-context data	
	Qasrawi (2022) [40]	Prediction of depression and anxiety		Machine learning		Survey-based data	
	Kamińska (2023) [41]	Classification of stress levels		Machine learning		Physiological data	
	Sahoo (2024) [24]	Prediction of mental health (depressive and anxiety symptoms, hostility, and somatization)		Machine learning		Survey-based data	
	Dell (2024) [43]	Classification of depression and PTSD		Machine learning		Survey-based data	
	So (2024) [44]	Identification of psychiatric symptoms (symptoms of PTSD, complex PTSD, depressive disorders, anxiety disorders, and alcohol use disorders)		Large language models		Interview transcripts	
	Saleh (2024) [45]	Prediction of depressive symptoms		Machine learning		Survey-based data	
	Bashar (2025) [48]	Classification of PTSD, depression, anxiety, and insomnia		Machine learning		Survey-based data	
	Habashneh (2025) [49]	Prediction of depression		Machine learning		Survey-based data	
	Figueiredo (2025) [17]	Prediction of PTSD		Machine learning		Survey-based data	
	Keller (2025) [50]	Prediction of suicidal ideation		Machine learning		Survey-based data	
	Im (2026) [52]	Prediction of depressive symptom severity		Machine learning		Survey-based data	
**Category 2: Intervention and Clinical Support**							
	Ugan (2023) [19]	Automated speech recognition and translation		Speech recognition, machine translation, speech synthesis		Multilingual speech corpora and psychiatric interview recordings	
	Pozzi (2024) [42]	Psychotherapy (mental health chatbot)		Natural language processing		Not specified	
	Gellert (2025) [51]	Automated symptom evaluation and referral		AI (not specified)		Not specified	
	Demetry (2026) [53]	Psychotherapy (cognitive behavioral therapy)		Large language models		Not specified	
**Category 3. Information Synthesis**							
	Khatua (2021) [23]	Concern identification and thematic classification		Deep learning		Tweets	
	So (2024) [44]	Symptom summarization		Large language models		Interview transcripts	
	Shrivastava (2025) [46]	Insight extraction		Large language models		Unstructured refugee health documents	
	Ashqar (2025) [47]	Sentiment analysis and summarization		Natural language processing, large language models		Oral testimonies	
	Khatua (2025) [25]	Summarization, simulation, and policy generation		Large language models		Tweets	

^a^PTSD: posttraumatic stress disorder.

First, studies in which AI was primarily used to detect, predict, or classify mental health outcomes—or to incorporate refugee mental health variables into predictive models—were classified into the Detection, Prediction, and Classification category. This was the largest category, representing 65.2% (n=15) of the included studies. Within this group, 14 of the 15 studies used machine learning models to assess mental health outcomes such as PTSD, depression, and suicidal ideation. While most of these studies relied on survey data as model inputs, nontraditional data sources were also explored: 2 studies used children’s drawings [18,39], 1 study analyzed audio data [38], and 1 study incorporated physiological data, specifically electroencephalogram and galvanic skin conductance [41]. Additionally, 1 study leveraged LLMs to extract psychiatric symptoms directly from the interview transcripts [44].

Second, studies in which AI was primarily used to deliver mental health interventions or support clinical care were classified into the Intervention and Clinical Support category, which comprises 4 (17.4%) studies. This category focused on clinical applications, including an AI-based virtual triage and care referral platform for automated symptom evaluation [51], an AI-based mental health chatbot [42], AI-based culturally adapted cognitive behavioral therapy [53], and a speech-to-speech translation tool that uses automatic speech recognition and machine translation to bridge linguistic barriers for refugees [19].

Third, studies in which AI was primarily used to extract and synthesize various sources of data regarding refugee mental health through AI-based techniques such as summarization, sentiment analysis, and topic modeling were classified into the Information Synthesis category, which included 5 (21.7%) studies. The majority (n=4) used LLMs to summarize complex, unstructured information from sources such as interview transcripts, health documents, video interviews, and tweets [25,44,46,47]. One remaining study used deep learning approaches to analyze tweets and identify concerns and struggles of refugees [23]. Notably, 1 study further used these summarized data as an input for agent-based simulations to generate policy recommendations [25].

One study that used LLMs to summarize stressors and symptoms and to identify estimated symptoms from interview transcripts was classified into both the Detection, Prediction, and Classification and the Information Synthesis categories [44]. Accordingly, the functional categories were not mutually exclusive.

### Mental Health Conditions of Interest

Among the 23 studies included in the review, 18 addressed specific mental health conditions, while five focused on the broader technical or ethical infrastructure of AI in refugee mental health without targeting a specific condition [19,42,46,47,53]. The remaining studies targeted a diverse range of conditions, which can be categorized into (1) diagnostic mental health symptoms, (2) affective and behavioral symptoms, and (3) global distress and social outcomes.

Among diagnostic mental health symptoms, depression was the most frequently examined condition, identified in over half of the studies (n=14; [24,25,37-40,43-45,48-52]), followed by PTSD (n=9; [17,18,37-39,43,44,48,50]) with 1 study specifically examining complex PTSD [44]. Anxiety was also addressed in one-third of the included studies (n=8; [24,25,39,40,43,44,48,51]). Other mental health conditions included insomnia (n=2; [48,51]), somatization (n=2; [24,51]), and alcohol use disorder (n=1; [44]).

Affective and behavioral symptoms included suicidal thoughts (n=2; [50,51]), guilt (n=1; [52]), and hostility (n=1; [24]). Notably, Gellert et al [51] used AI to screen for a broad spectrum of behavioral indicators, including irritability, nervousness, agitation, and fear of a specific object, situation, or action.

Lastly, several studies targeted broader psychological and social constructs relevant to refugee mental health. These included trauma and associated sufferings (n=2; [23,39]), psychological distress (n=2; [25,39]), and physiological stress (n=1; [41]).

### Potential Strengths of AI-Based Approaches to Refugee Mental Health Care

In the Detection, Prediction, and Classification category, the included studies suggested several potential strengths, including accessibility and scalability in resource-limited settings and improved clinical workflows. AI may provide low-threshold access to identify vulnerable groups, such as refugee minors [38,49,50], using cost-effective and noninvasive methods such as the analysis of hand drawings [18,39]. These scalable methods were described as potentially helpful where mental health needs exceed available resources [24,44] and culturally sensitive diagnostics are needed [48]. Furthermore, these approaches may facilitate early prediction and examination of mental health symptoms [38,40] and potentially enhance clinical workflows through decision support, automated assessment of symptoms, and intervention planning [17,41,44].

Strengths in the Intervention and Clinical Support primarily related to overcoming traditional barriers to care. AI-based systems may mitigate language barriers in conflict settings, reducing the cost and preventing refugees’ hesitance to share experiences with human interpreters due to stigma [19]. Additionally, virtual triage systems were shown to enable the monitoring of population needs, facilitating early detection and intervention in refugees and displaced persons [51].

Studies classified in the Information Synthesis category reported a structured approach to analyzing unstructured data, generating actionable policy insights, and enhancing clinical workflow as potential strengths of AI-based approaches [23,25,44,47].

### Reported Limitations of AI-Based Approaches to Refugee Mental Health Care

The majority of studies in the Detection, Prediction, and Classification category focused on technical and methodological constraints rather than context-specific limitations. A primary concern was the use of small sample sizes in machine learning models, which may result in higher error rates and diminished model robustness [17,37,38,43]. Beyond technical constraints, several studies cautioned against an uncritical reliance on AI. Dell et al [43] identified automation bias, wherein practitioners may uncritically accept AI-generated results despite having sufficient knowledge and training.

In the Intervention and Clinical Support category, studies highlighted barriers to equitable use and ethical design. For the AI-based virtual triage platform, reliance on digital literacy and technology access was reported as a significant barrier to equity [51]. Regarding conversational AI (CAI) for psychotherapy, Pozzi and De Proost [42] argued that these systems often oversimplify the therapeutic process, as they are currently insufficient to facilitate epistemically richer interactions—such as self-understanding, hypothesizing, and critical analysis—required in the therapy process. This may be further complicated by identity bias, where the imposition of Western psychotherapeutic frameworks onto non-Western populations may create a cultural mismatch [42]. Finally, limitations in algorithm development may also include the neglect of refugee agency, where individuals are treated as passive participants rather than active contributors to the CAI design.

In the Information Synthesis category, limitations primarily concerned the loss of emotional depth and narrative integrity. While LLMs may be helpful in processing large volumes of data, they are often limited in capturing the full emotional complexity of traumatic experiences [47]. Furthermore, So et al [44] noted that heavy reliance on LLMs for clinical summarization could undermine therapeutic relationships by bypassing the rapport-building essential to face-to-face care. Lastly, Khatua and Nejdl [23] identified a visibility bias, as refugees in asylum centers often lack internet connectivity required to share their experiences online, rendering them invisible in social media–based synthesis.

### Suggested Future Directions of AI-Based Approaches to Refugee Mental Health Care

The Detection, Prediction, and Classification category yielded the most recommendations for future research, which can be categorized into three primary themes: (1) data enrichment, (2) development of robust decision-making guidelines, and (3) translational efforts. First, several studies underscored the necessity of larger and more comprehensive datasets to improve diagnostic accuracy and evaluate clinical use [38,43]. Alternatively, some studies suggested focusing on specific subpopulations to refine model training [18,38] or incorporating a broad range of variables—such as biomarkers, cultural factors, and multimodal assessments—to develop more robust predictive models [43,48,50]. Second, the literature highlighted the critical need for robust guidelines to govern the translation of AI-based outcomes into clinical and policy decision-making. Figueiredo and Ndiaye [17] argued that the final authority in decision-making should remain with humans, who are better equipped to understand contextual nuances and subjective experiences of refugees. Similarly, Keller et al [50] noted that the extent to which AI can influence decision-making should be determined. Third, regarding translational efforts, Figueiredo and Ndiaye [17] emphasized the need for specialized training to help practitioners accurately interpret and use predictive models in real-world settings.

In the Intervention and Clinical Support category, future directions focused on clinical validation and human-centered design. Gellert et al [51] noted that AI-based virtual triage and care referral platforms introduced in the study lack clinical validation, warranting future investigation. Furthermore, Pozzi and De Proost [42] argued that CAI users should be treated as active participants of interaction for mental health support, rather than objects, necessitating context-sensitive designs that incorporate the target population’s cultural diversity and societal values.

Similarly, within the Information Synthesis category, Ashqar [47] called for the development of specialized models tailored to specific target populations. This may involve creating systems capable of understanding the unique cultural and historical contexts of displaced groups to accurately convey the complexity and emotional depth of the narratives.

### Integrated Summary of AI-Based Approaches to Refugee Mental Health Care

Table 3 provides an integrated summary of strengths, limitations, and future directions identified across 3 functional categories of AI-based approaches to refugee mental health care. Overall, the included studies reported accessibility, scalability, and the ability to synthesize complex data as strengths of AI-based approaches, while important concerns remained regarding biases, technology access, and lack of contextual understanding. Future directions commonly emphasized the need for translational research, clinical validation, and human-centered development to support ethical and practical integration of AI into refugee mental health care.

**Table 3:** Integrated summary of the reported strengths, limitations, and future directions of AI-based approaches to refugee mental health care.

	Strengths	Limitations	Future directions
Detection, Prediction, and Classification	Accessibility and scalability in resource-limited settings Improved clinical workflows	Small sample sizes Algorithmic bias Automation bias	Data enrichment Development of robust decision-making guidelines Translational efforts
Intervention and Clinical Support	Overcoming traditional barriers to care (eg, language barrier) Early detection and intervention	Need for digital literacy and technology access Lack of epistemically richer interactions Identity bias Neglect of refugee agency	Clinical validation of AI-based systems Human-centered design
Information Synthesis	Structured analysis of unstructured data (eg, tweets)	Loss of emotional depth Possibility of therapeutic relationships undermined	Development of specialized models for specific populations

## Discussion

### Principal Findings

In this systematic integrative review, we identified 23 studies on AI-based approaches to refugee mental health care. Specifically, we classified the use of AI into three functional categories: (1) Detection, Prediction, and Classification; (2) Intervention and Clinical Support; and (3) Information Synthesis. Furthermore, we identified strengths, limitations, and future directions of AI use in refugee mental health care across three functional categories. To our knowledge, this is the first review to systematically identify and evaluate the use of AI primarily in refugee mental health care. This review is particularly timely given the rapid advancement of AI technologies, as reflected in the increasing number of studies identified over time, alongside the unprecedented global displacement of refugees and forcibly displaced populations [1].

While an inductive approach was used to generate functional categories of the included studies, the resulting classification is broadly consistent with previous reviews of AI in mental health care, which identified AI applications in diagnosis, monitoring, intervention, and mental health support [10,11]. These functions largely correspond to the Detection, Prediction, and Classification and the Intervention and Clinical Support categories identified in the present review. In contrast, Information Synthesis did not emerge as a distinct functional category in previous reviews, likely because they primarily focused on AI applications in individual-level mental health care. By adopting a broader perspective on refugee mental health, the present review identified studies that used AI to generate population-level insights, such as extracting information from unstructured refugee datasets and synthesizing refugees’ experiences and mental health concerns expressed on social media, which resulted in the distinct category of Information Synthesis.

The most prominent use of AI in refugee mental health care was the detection of mental health and psychiatric symptoms, consistent with a previous review on AI in mental health care reporting a majority of studies classified as diagnosis and monitoring domains [10]. By facilitating low-threshold identification of individuals vulnerable to mental health problems, AI-based systems may be particularly useful in resource-limited humanitarian settings [38,40,49,50]. Several studies have used innovative approaches that used hand drawings as input data [18,39], demonstrating potential use among children and individuals facing language barriers. However, the predominance of detection-focused studies relative to intervention- and policy-oriented applications suggests that AI research in refugee mental health remains concentrated on predictive modeling rather than delivery, implementation, or evaluation of real-world solutions. Future research should examine how to translate these predictive models into AI tools that can be deployed in real-world humanitarian settings for rapid identification of psychiatric symptoms, efficient resource allocation, and clinical decision-making.

The small number of studies identified within the Intervention and Clinical Support category highlights a substantial gap in the current literature. While accurate detection is important, improvements in refugee mental health may ultimately depend on the availability and effectiveness of interventions that can be delivered following identification of need. Although AI-based psychotherapies, including conversational agents and game-based interventions, have been developed and evaluated in nonrefugee populations [54,55], comparable applications remain largely understudied among refugees. This gap is particularly important because existing AI systems may not generalize well to refugee populations, given their predominant development using Western datasets and the unique linguistic, cultural, and contextual challenges experienced by refugees [26,27,56]. Consistent with broader critiques of humanitarian technologies, implementation barriers such as limited internet access, mobile device availability, and language accessibility may further limit equitable access to these interventions [57]. Such concerns raise the possibility that AI-based interventions could inadvertently reproduce existing inequalities unless refugees are meaningfully involved in their development and implementation [57].

Accordingly, beyond addressing potential biases embedded within AI systems, the development of AI-based interventions should not merely be conducted for refugees but in partnership with refugees. Pozzi and De Proost [42] identified the neglect of refugee agency as a key limitation in the design of CAI interventions, highlighting the importance of participatory approaches in intervention development. Similarly, Demetry et al [53] actively involved refugees in evaluating the cultural relevance and acceptability of cognitive behavioral therapy culturally adapted by AI vs human psychologists. Meaningful involvement of refugees throughout the design and evaluation process may help ensure that interventions align with users’ needs, experiences, and cultural contexts. Co-designed interventions that recognize refugees as active contributors rather than passive recipients may ultimately improve the relevance, acceptability, and effectiveness of AI-based mental health support.

Within the Information Synthesis category, AI-based approaches—the majority of which were LLMs—demonstrated potential for processing and summarizing large volumes of complex and unstructured refugee-related data. Such capabilities may help researchers, clinicians, and policymakers identify patterns and generate insights from information that would otherwise be difficult to synthesize. However, the value of AI-based information synthesis depends not only on efficiency but also on the accuracy of how refugee experiences are represented. Existing studies have highlighted concerns that AI-generated summaries may inadequately capture the emotional complexity and contextual nuance embedded in refugee narratives [47] and may introduce visibility bias by underrepresenting refugees with limited digital access or literacy [23]. Furthermore, although not discussed in the included studies, generative AI may produce and disseminate plausible but inaccurate representations of refugee experiences, particularly when trained on biased or inaccurate information [58-60]. Such inaccuracies may indirectly contribute to refugee mental health by reinforcing misinformation, stigma, and hostile social attitudes toward refugee populations [61]. Accordingly, future research should evaluate not only the efficiency of AI-based information synthesis but also its ability to accurately represent the experiences and perspectives of diverse refugee populations.

Notably, more than one-third of the included studies focused specifically on child and adolescent refugees, with all but one study falling within the Detection, Prediction, and Classification category. Several studies highlighted the potential strengths of AI-based approaches for this subgroup, including noninvasive, low-threshold methods for identifying mental health conditions among children and adolescents [18,38,39]. However, none of these studies explicitly addressed the unique ethical, methodological, or clinical considerations associated with applying AI to refugee children and adolescents. This gap is particularly noteworthy given that many children are separated from their families during displacement and must navigate migration-related challenges alone, as reflected by the estimated 153,300 unaccompanied and separated children worldwide [62,63]. Compared with accompanied children and the general population of the same age, unaccompanied refugee children experience higher levels of psychiatric distress, including PTSD and suicidal behaviors [64,65]. Moreover, trauma exposure during childhood and adolescence may have long-term developmental consequences [66,67]. Despite these distinct vulnerabilities, included studies lack considerations tailored to this subgroup. Core ethical principles of beneficence and nonmaleficence may be especially important [68] when developing and implementing AI systems for individuals occupying the intersectional position of being both a child and a forcibly displaced person. Given that many AI applications rely on highly sensitive data, including psychiatric and trauma-related data [18,38-40,45,49,50], robust safeguards are needed to protect privacy, promote well-being, and minimize potential harms.

More broadly, several ethical considerations received limited attention across the included studies despite their importance for the responsible use of AI in refugee mental health care. First, privacy and confidentiality are particularly sensitive concerns in refugee populations, given that misuse of personal information may expose individuals to substantial threats [58]. The International Committee of the Red Cross and Privacy International have highlighted the humanitarian metadata problem, whereby seemingly innocuous metadata can be combined with other sources of information to reveal sensitive behavioral patterns and facilitate profiling and surveillance [69]. These concerns are particularly relevant in refugee contexts, where digital technologies may simultaneously facilitate access to services while increasing the potential for monitoring, identification, and control throughout the displacement process [70]. Consequently, even AI systems developed with humanitarian intentions may inadvertently expose refugees to harm if appropriate safeguards are not in place, raising important concerns regarding the ethical principle of nonmaleficence [69]. Especially among individuals with uncertain legal status, AI systems may facilitate identification, tracking, or surveillance, potentially increasing vulnerability to detention, exploitation, and deportation [71]. Such experiences may, in turn, contribute to psychological distress and adverse mental health outcomes [72,73].

Second, meaningful inclusion of refugee perspectives throughout the AI life cycle warrants greater attention. Beyond intervention development [42,53], meaningful refugee participation should also extend to data collection, model development, implementation, and the composition of training datasets. Meaningful participation requires diverse representation of refugee populations, including individuals facing barriers to engagement, while remaining attentive to power imbalances and the need to foster safe and trustworthy environments for collaboration [74]. In addition, refugee representation should be considered at the level of training data. Given that many existing AI models have been developed using datasets derived predominantly from Western, educated, industrialized, rich, and democratic populations [26,27], inadequate representation of refugee experiences may introduce biases that limit the relevance of AI-generated outputs. This concern echoes broader critiques of humanitarian technologies, which caution that systems developed without meaningful participation from refugees and other affected populations may inadvertently reproduce existing inequities and reinforce power asymmetries [75]. Although privacy, confidentiality, and refugee participation do not constitute an exhaustive list of ethical considerations, these issues warrant careful attention in future research and implementation efforts involving AI-based approaches to refugee mental health care.

### Limitations

This review has several limitations. First, AI is a rapidly evolving field, and new studies continue to emerge. Although literature searches were conducted at two time points (December 2025 and June 2026) to capture the most recent evidence, studies published after June 2026 were not included.

Second, despite conducting systematic literature searches using comprehensive search terms informed by previous literature and reviewed by a medical librarian, relevant studies may have been missed, particularly those using emerging AI models or unconventional terminology. To maximize comprehensiveness, supplementary searches were conducted in Google Scholar; however, the platform’s nontransparent and dynamic relevance-ranking algorithm may limit reproducibility. Nevertheless, Google Scholar was used only as a supplementary search source, and a wide range of bibliographic databases were searched. Additionally, although gray literature (eg, reports from humanitarian organizations) may contain valuable information regarding AI use in refugee contexts, this review focused on scholarly literature. Consequently, relevant nonacademic evidence may not be reflected in the findings.

Third, this review adopted a broad conceptualization of forcibly displaced populations—encompassing statutory refugees, internally displaced people, asylum seekers, and individuals displaced by climate-related disasters—regardless of formal legal status. While these populations share experiences related to forced displacement, trauma exposure, and mental health vulnerability [6,7,33,34], such broad inclusion may introduce population heterogeneity. Furthermore, distinctions between forcibly displaced populations and broader migrant populations were not clear in several studies, requiring case-by-case eligibility decisions based on predefined strict criteria and team discussion.

Fourth, only a small number of studies were identified within the Intervention and Clinical Support and the Information Synthesis categories. Furthermore, the included studies were conducted across diverse refugee and forcibly displaced populations, settings, and contexts. Consequently, findings presented in this review should be interpreted as preliminary and may not be generalizable across refugee populations and humanitarian settings. Additionally, one of the included studies was a non–peer-reviewed preprint; thus, it is possible that findings derived from this study may differ from the eventual published version.

Fifth, although quality appraisal was conducted in accordance with integrative review methodology, no widely accepted appraisal tool currently exists for the evaluation of engineering- and computing-focused studies. Accordingly, formal quality appraisal was restricted to empirical health research studies, while studies primarily focused on AI system development, optimization, or technical performance evaluation were not formally appraised. Consequently, the quality appraisal findings presented in this review should be interpreted only for the studies that underwent formal appraisal and should not be generalized to engineering- and computing-focused studies.

Sixth, the integrative review allows for the inclusion of diverse study designs and evidence types [30], which was essential for comprehensively mapping this emerging field. However, heterogeneity may limit the degree to which findings can be synthesized using more structured or standardized approaches. As the field matures, future reviews may benefit from narrower review questions and methodologies, such as systematic reviews or meta-analyses, to evaluate specific applications of AI in refugee mental health care.

Finally, while this review identified the limited inclusion of refugee perspectives as an important concern within the existing literature, the review itself was conducted without direct involvement of refugees or forcibly displaced persons. Future research may benefit from incorporating refugee perspectives not only into AI system development but also into evidence synthesis processes.

### Conclusion

This systematic integrative review is, to our knowledge, the first to comprehensively synthesize AI-based approaches specifically for refugee mental health care. Unlike previous reviews that have focused primarily on digital mental health interventions or AI applications in general populations, this review focused on refugee populations who experience unique mental health challenges and face heightened concerns regarding privacy and confidentiality. This review identified three functional domains that characterize the current landscape of AI applications in refugee mental health care, including symptom detection, clinical support, and information synthesis. By synthesizing current applications alongside their methodological, ethical, and implementation challenges, this review provides a foundation for future research and offers practical considerations for researchers, clinicians, humanitarian organizations, and policymakers seeking to ethically and contextually integrate AI into refugee mental health care.

## Data Availability

Data sharing is not applicable to this article as no datasets were generated or analyzed during this study.

## Appendix

Multimedia Appendix 1 PRISMA 2020 checklist.

Multimedia Appendix 2 PRISMA 2020 for Abstracts checklist.

Multimedia Appendix 3 PRISMA-S checklist.

Multimedia Appendix 4 Search strategy for each database.

Multimedia Appendix 5 Results of quality appraisal.

## Abbreviations

CAI: conversational AI
JBI: Joanna Briggs Institute
LLM: large language model
MMAT: Mixed Methods Appraisal Tool
PRISMA: Preferred Reporting Items for Systematic Reviews and Meta-Analyses
PRISMA-S: Preferred Reporting Items for Systematic Reviews and Meta-Analyses literature search extension
PROSPERO: International Prospective Register of Systematic Reviews
PTSD: posttraumatic stress disorder
