# Supplementary material for: AI-Based Approaches to Refugee Mental Health Care: Systematic Integrative Review
Source: J Med Internet Res. 2026 Aug 26;28:e101857. doi: 10.2196/101857 (PMC13559157; doi:10.2196/101857)
Supplement: Supplementary file 4 [file jmir_v28i1e101857_app4.docx]

**Multimedia Appendix 4.** Search strategy for each database

1. PubMed (search date: December 5, 2025 & June 18, 2026)

| #1 | refugee*[tiab] OR "asylum seeker*"[tiab] OR asylee*[tiab] OR "displaced population*"[tiab] OR "displaced person*"[tiab] OR "internally displaced"[tiab] OR IDPs[tiab] OR IDP[tiab] OR defector*[tiab] OR "humanitarian migrant*"[tiab] OR "forced migration"[tiab] OR "forced displacement"[tiab] OR "forcibly displaced"[tiab] OR "forced migrant*"[tiab] OR immigration[tiab] OR immigrant*[tiab] OR migrant*[tiab] OR "Emigrants and Immigrants"[MeSH] OR "Emigration and Immigration" OR "Refugees"[MeSH] |
| --- | --- |
| #2 | "artificial intelligence*"[tiab] OR AI[tiab] OR "large language model*"[tiab] OR LLM[tiab] OR "natural language processing"[tiab] OR NLP[tiab] OR "machine learning"[tiab] OR "deep learning"[tiab] OR "supervised learning"[tiab] OR "unsupervised learning"[tiab] OR "neural network*"[tiab] OR "digital phenotyping"[tiab] OR "predictive model*"[tiab] OR chatbot[tiab] OR "rule-based"[tiab] OR robotics[tiab] OR "computer vision"[tiab] OR "image generator*"[tiab] OR "Artificial Intelligence"[MeSH] |
| #3 | mental[tiab] OR psychiatric[tiab] OR psychosocial[tiab] OR psychological[tiab] OR cognitive[tiab] OR depress*[tiab] OR anxiety[tiab] OR PTSD[tiab] OR trauma[tiab] OR stress[tiab] OR distress[tiab] OR "posttraumatic stress"[tiab] OR "post-traumatic stress"[tiab] OR suicid*[tiab] OR bipolar[tiab] OR sleep[tiab] OR schizophrenia[tiab] OR psychosis[tiab] OR "obsessive compulsive disorder"[tiab] OR "mood disorder"[tiab] OR "memory disorder"[tiab] OR  "attention deficit hyperactivity disorder"[tiab] OR "affective disorder"[tiab] OR "stress-related disorder"[tiab] OR  "Mental Health"[MeSH] OR "Mental Disorders"[MeSH] |
| #4 | #1 AND #2 AND #3 |

2. EMBASE (search date: December 5, 2025 & June 18, 2026)

| #1 | refugee*:ti,ab,kw OR "asylum seeker*":ti,ab,kw OR asylee*:ti,ab,kw OR "displaced population*":ti,ab,kw OR "displaced person*":ti,ab,kw OR "internally displaced":ti,ab,kw OR IDPs:ti,ab,kw OR IDP:ti,ab,kw OR defector*:ti,ab,kw OR "humanitarian migrant*":ti,ab,kw OR "forced migration":ti,ab,kw OR "forced displacement":ti,ab,kw OR "forcibly displaced":ti,ab,kw OR "forced migrant*":ti,ab,kw OR immigration:ti,ab,kw OR immigrant*:ti,ab,kw OR migrant*:ti,ab,kw OR refugee/exp OR "displaced person"/exp OR migrant/exp OR migration/exp |
| --- | --- |
| #2 | "artificial intelligence*":ti,ab,kw OR AI:ti,ab,kw OR "large language model*":ti,ab,kw OR LLM:ti,ab,kw OR "natural language processing":ti,ab,kw OR NLP:ti,ab,kw OR "machine learning":ti,ab,kw OR "deep learning":ti,ab,kw OR "supervised learning":ti,ab,kw OR "unsupervised learning":ti,ab,kw OR "neural network*":ti,ab,kw OR "digital phenotyping":ti,ab,kw OR "predictive model*":ti,ab,kw OR chatbot:ti,ab,kw OR "rule-based":ti,ab,kw OR robotics:ti,ab,kw OR "computer vision":ti,ab,kw OR "image generator*":ti,ab,kw OR "artificial intelligence"/exp OR "machine learning"/exp OR "natural language processing"/exp OR "deep learning"/exp OR "large language model"/exp |
| #3 | mental:ti,ab,kw OR psychiatric:ti,ab,kw OR psychosocial:ti,ab,kw OR psychological:ti,ab,kw OR cognitive:ti,ab,kw OR depress*:ti,ab,kw OR anxiety:ti,ab,kw OR PTSD:ti,ab,kw OR trauma:ti,ab,kw OR stress:ti,ab,kw OR distress:ti,ab,kw OR "posttraumatic stress":ti,ab,kw OR "post-traumatic stress":ti,ab,kw OR suicid*:ti,ab,kw OR bipolar:ti,ab,kw OR sleep:ti,ab,kw OR schizophrenia:ti,ab,kw OR psychosis:ti,ab,kw OR "obsessive compulsive disorder":ti,ab,kw OR "mood disorder":ti,ab,kw OR "memory disorder":ti,ab,kw OR "attention deficit hyperactivity disorder":ti,ab,kw OR "affective disorder":ti,ab,kw OR "stress-related disorder":ti,ab,kw OR "mental health"/exp OR "mental disease"/exp |
| #4 | #1 AND #2 AND #3 |

3. CINAHL (search date: December 5, 2025 & June 18, 2026)

| S1 | XB(refugee* OR "asylum seeker*" OR asylee* OR "displaced person*" OR "displaced population*" OR "internally displaced" OR IDP* OR defector* OR "humanitarian migrant*" OR "forced migration" OR "forced displacement" OR "forcibly displaced" OR immigration OR immigrant* OR migrant* OR "forced migrant*" ) OR (MH "Refugees+" OR MH "Immigrants+" OR MH "Emigration and Immigration+" OR MH "Migrant") |
| --- | --- |
| S2 | XB("artificial intelligence*" OR AI OR "large language model*" OR LLM OR "natural language processing" OR NLP OR "machine learning" OR "deep learning" OR "supervised learning" OR "unsupervised learning" OR "neural network*" OR "digital phenotyping" OR "predictive model*" OR chatbot OR "rule-based" OR robotics OR "computer vision" OR "image generator*") OR (MH "Artificial Intelligence+") |
| S3 | XB(mental OR psychiatric OR psychosocial OR psychological OR cognitive OR depress* OR anxiety OR PTSD OR trauma OR stress OR distress OR "posttraumatic stress" OR "post-traumatic stress" OR suicid* OR bipolar OR sleep OR schizophrenia OR psychosis OR "obsessive compulsive disorder" OR "mood disorder" OR "memory disorder" OR "attention deficit hyperactivity disorder" OR "affective disorder" OR "stress-related disorder") OR (MH "Mental Health+" OR MH "Behavioral and Mental Disorders+") |
| S4 | S1 AND S2 AND S3 |

4. PsycINFO (search date: December 5, 2025 & June 18, 2026)

| S1 | tiab((refugee* OR "asylum seeker*" OR asylee* OR "displaced person*" OR "displaced population*" OR "internally displaced" OR IDP* OR defector* OR "humanitarian migrant*" OR "forced migration" OR "forced displacement" OR "forcibly displaced" OR immigration OR immigrant* OR migrant* OR "forced migrant*") OR (DE "Refugees" OR DE "Asylum Seeking+" OR DE "Human Migration+")) |
| --- | --- |
| S2 | tiab(("artificial intelligence*" OR AI OR "large language model*" OR LLM OR "natural language processing" OR NLP OR "machine learning" OR "deep learning" OR "supervised learning" OR "unsupervised learning" OR "neural network*" OR "digital phenotyping" OR "predictive model*" OR chatbot OR "rule-based" OR robotics OR "computer vision" OR "image generator*") OR (DE "Artificial Intelligence+")) |
| S3 | tiab((mental OR psychiatric OR psychosocial OR psychological OR cognitive OR depress* OR anxiety OR PTSD OR trauma OR stress OR distress OR "posttraumatic stress" OR "post-traumatic stress" OR suicid* OR bipolar OR sleep OR schizophrenia OR psychosis OR "obsessive compulsive disorder" OR "mood disorder" OR "memory disorder" OR "attention deficit hyperactivity disorder" OR "affective disorder" OR "stress-related disorder") OR (DE "Mental Health" OR DE "Mental Disorders")) |
| S4 | [S1] AND [S2] AND [S3] |

5. Scopus (search date: December 5, 2025 & June 18, 2026)

| S1 | TITLE-ABS-KEY ( refugee* OR "asylum seeker*" OR asylee* OR "displaced person*" OR "displaced population*" OR "internally displaced" OR IDP* OR defector* OR "humanitarian migrant*" OR "forced migration" OR "forced displacement" OR "forcibly displaced" OR immigration OR immigrant* OR migrant* OR "forced migrant*" ) |
| --- | --- |
| S2 | TITLE-ABS-KEY ( "artificial intelligence*" OR AI OR "large language model*" OR LLM OR "natural language processing" OR NLP OR "machine learning" OR "deep learning" OR "supervised learning" OR "unsupervised learning" OR "neural network*" OR "digital phenotyping" OR "predictive model*" OR chatbot* OR "rule-based" OR robotics OR "computer vision" OR "image generator*" ) |
| S3 | TITLE-ABS-KEY ( mental OR psychiatric OR psychosocial OR psychological OR cognitive OR depress* OR anxiety OR PTSD OR trauma OR stress OR distress OR "posttraumatic stress" OR "post-traumatic stress" OR suicid* OR bipolar OR sleep OR schizophrenia OR psychosis OR "obsessive compulsive disorder" OR "mood disorder" OR "memory disorder" OR "attention deficit hyperactivity disorder" OR "affective disorder" OR "stress-related disorder" ) |
| S4 | S1 AND S2 AND S3 |

6. Web of Science (search date: December 5, 2025 & June 18, 2026)

| S1 | **TS**=(refugee* OR "asylum seeker*" OR asylee* OR "displaced person*" OR "displaced population*" OR "internally displaced" OR IDP* OR defector* OR "humanitarian migrant*" OR "forced migration" OR "forced displacement" OR "forcibly displaced" OR immigration OR immigrant* OR migrant* OR "forced migrant*") |
| --- | --- |
| S2 | TS=("artificial intelligence*" OR AI OR "large language model*" OR LLM OR "natural language processing" OR NLP OR "machine learning" OR "deep learning" OR "supervised learning" OR "unsupervised learning" OR "neural network*" OR "digital phenotyping" OR "predictive model*" OR chatbot* OR "rule-based" OR robotics OR "computer vision" OR "image generator*") |
| S3 | TS=(mental OR psychiatric OR psychosocial OR psychological OR cognitive OR depress* OR anxiety OR PTSD OR trauma OR stress OR distress OR "posttraumatic stress" OR "post-traumatic stress" OR suicid* OR bipolar OR sleep OR schizophrenia OR psychosis OR "obsessive compulsive disorder" OR "mood disorder" OR "memory disorder" OR "attention deficit hyperactivity disorder" OR "affective disorder" OR "stress-related disorder") |
| S4 | S1 AND S2 AND S3 |

7. Cochrane Library (search date: December 5, 2025 & June 18, 2026)

| #1 | (refugee* OR (asylum NEXT seeker*) OR asylee* OR (displaced NEXT person*) OR (displaced NEXT population*) OR "internally displaced" OR IDP* OR defector* OR (humanitarian NEXT migrant*) OR "forced migration" OR "forced displacement" OR "forcibly displaced" OR immigration OR immigrant* OR migrant* OR (forced NEXT migrant*)):ti,ab,kw OR [mh "Refugees"] OR [mh "Emigrants and Immigrants"] OR [mh "Emigration and Immigration"] |
| --- | --- |
| #2 | ((artificial NEXT intelligence*) OR AI OR (large NEXT language NEXT model*) OR LLM OR "natural language processing" OR NLP OR "machine learning" OR "deep learning" OR "supervised learning" OR "unsupervised learning" OR (neural NEXT network*) OR "digital phenotyping" OR (predictive NEXT model*) OR chatbot* OR "rule-based" OR robotics OR "computer vision" OR (image NEXT generator*)):ti,ab,kw OR [mh "Artificial Intelligence"] |
| #3 | (mental OR psychiatric OR psychosocial OR psychological OR cognitive OR depress* OR anxiety OR PTSD OR trauma OR stress OR distress OR "posttraumatic stress" OR "post-traumatic stress" OR suicid* OR bipolar OR sleep OR schizophrenia OR psychosis OR "obsessive compulsive disorder" OR "mood disorder" OR "memory disorder" OR "attention deficit hyperactivity disorder" OR "affective disorder" OR "stress-related disorder"):ti,ab,kw OR [mh "Mental Health"] OR [mh "Mental Disorders"] |
| #4 | #1 AND #2 AND #3 |

8. The ACM Guide to Computing Literature (search date: December 5, 2025 & June 18, 2026)

| #1 | Abstract:(refugee* OR "asylum seeker*" OR asylee* OR "displaced person*" OR "displaced population*" OR "internally displaced" OR IDP* OR defector* OR "humanitarian migrant*" OR "forced migration" OR "forced displacement" OR "forcibly displaced" OR immigration OR immigrant* OR migrant* OR "forced migrant*") OR Title:(refugee* OR "asylum seeker*" OR asylee* OR "displaced person*" OR "displaced population*" OR "internally displaced" OR IDP* OR defector* OR "humanitarian migrant*" OR "forced migration" OR "forced displacement" OR "forcibly displaced" OR immigration OR immigrant* OR migrant* OR "forced migrant*") |
| --- | --- |
| #2 | Abstract:("artificial intelligence*" OR AI OR "large language model*" OR LLM OR "natural language processing" OR NLP OR "machine learning" OR "deep learning" OR "supervised learning" OR "unsupervised learning" OR "neural network*" OR "digital phenotyping" OR "predictive model*" OR chatbot* OR "rule-based" OR robotics OR "computer vision" OR "image generator*") OR Title:("artificial intelligence*" OR AI OR "large language model*" OR LLM OR "natural language processing" OR NLP OR "machine learning" OR "deep learning" OR "supervised learning" OR "unsupervised learning" OR "neural network*" OR "digital phenotyping" OR "predictive model*" OR chatbot* OR "rule-based" OR robotics OR "computer vision" OR "image generator*") |
| #3 | Abstract:(mental OR psychiatric OR psychosocial OR psychological OR cognitive OR depress* OR anxiety OR PTSD OR trauma OR stress OR distress OR "posttraumatic stress" OR "post-traumatic stress" OR suicid* OR bipolar OR sleep OR schizophrenia OR psychosis OR "obsessive compulsive disorder" OR "mood disorder" OR "memory disorder" OR "attention deficit hyperactivity disorder" OR "affective disorder" OR "stress-related disorder") OR Title:(mental OR psychiatric OR psychosocial OR psychological OR cognitive OR depress* OR anxiety OR PTSD OR trauma OR stress OR distress OR "posttraumatic stress" OR "post-traumatic stress" OR suicid* OR bipolar OR sleep OR schizophrenia OR psychosis OR "obsessive compulsive disorder" OR "mood disorder" OR "memory disorder" OR "attention deficit hyperactivity disorder" OR "affective disorder" OR "stress-related disorder") |
| #4 | #1 AND #2 AND #3 |

9. IEEE Xplore (search date: December 5, 2025 & June 18, 2026)

| #1 | ("Document Title":refugee OR "asylum seeker" OR asylee OR "displaced person" OR "displaced population" OR "internally displaced" OR IDP* OR defector OR "humanitarian migrant" OR "forced migration" OR "forced displacement" OR "forcibly displaced" OR immigration OR immigrant OR migrant OR "forced migrant") OR ("Abstract":refugee OR "asylum seeker" OR asylee OR "displaced person" OR "displaced population" OR "internally displaced" OR IDP* OR defector OR "humanitarian migrant" OR "forced migration" OR "forced displacement" OR "forcibly displaced" OR immigration OR immigrant OR migrant OR "forced migrant") |
| --- | --- |
| #2 | ("Document Title":"artificial intelligence" OR AI OR "large language model" OR LLM OR "natural language processing" OR NLP OR "machine learning" OR "deep learning" OR "supervised learning" OR "unsupervised learning" OR "neural network" OR "digital phenotyping" OR "predictive model" OR chatbot OR "rule-based" OR robotics OR "computer vision" OR "image generator") OR ("Abstract":"artificial intelligence" OR AI OR "large language model" OR LLM OR "natural language processing" OR NLP OR "machine learning" OR "deep learning" OR "supervised learning" OR "unsupervised learning" OR "neural network" OR "digital phenotyping" OR "predictive model" OR chatbot OR "rule-based" OR robotics OR "computer vision" OR "image generator") |
| #3 | ("Document Title":mental OR psychiatric OR psychosocial OR psychological OR cognitive OR depressed OR depression OR depressive OR anxiety OR PTSD OR trauma OR stress OR distress OR "posttraumatic stress" OR "post-traumatic stress" OR suicide OR suicidal OR suicidality OR bipolar OR sleep OR schizophrenia OR psychosis OR "obsessive compulsive disorder" OR "mood disorder" OR "memory disorder" OR "attention deficit hyperactivity disorder" OR "affective disorder" OR "stress-related disorder") OR ("Abstract":mental OR psychiatric OR psychosocial OR psychological OR cognitive OR depressed OR depression OR depressive OR anxiety OR PTSD OR trauma OR stress OR distress OR "posttraumatic stress" OR "post-traumatic stress" OR suicide OR suicidal OR suicidality OR bipolar OR sleep OR schizophrenia OR psychosis OR "obsessive compulsive disorder" OR "mood disorder" OR "memory disorder" OR "attention deficit hyperactivity disorder" OR "affective disorder" OR "stress-related disorder") |
| #4 | #1 AND #2 AND #3 |

10. arXiv (search date: December 5, 2025 & June 18, 2026)

| #1 | "refugee" OR "refugees" OR "asylum seeker" OR "asylum seekers" OR "asylee" OR "asylees" OR "displaced population" OR "displaced populations" OR "displaced person" OR "displaced persons" OR "internally displaced" OR "IDP" OR "IDPs" OR "defector" OR "defectors" OR "humanitarian migrant" OR "humanitarian migrants" OR "forced migration" OR "forced displacement" OR "forcibly displaced" OR "forced migrant" OR "forced migrants" OR "immigration" OR "immigrant" OR "immigrants" OR "migrant" OR "migrants" |
| --- | --- |
| #2 | "artificial intelligence" OR "AI" OR "large language model" OR "large language models" OR "LLM" OR "natural language processing" OR "NLP" OR "machine learning" OR "deep learning" OR "supervised learning" OR "unsupervised learning" OR "neural network" OR "neural networks" OR "digital phenotyping" OR "predictive model" OR "predictive models" OR "chatbot" OR "algorithm" OR "algorithms" OR "rule-based" OR "robotics" OR "computer vision" OR "image generator" OR "image generators" |
| #3 | mental OR psychiatric OR psychosocial OR psychological OR cognitive OR depression OR depressive OR depressed OR anxiety OR PTSD OR trauma OR stress OR distress OR "posttraumatic stress" OR "post-traumatic stress" OR suicide OR suicidal OR suicidality OR bipolar OR sleep OR schizophrenia OR psychosis OR "obsessive compulsive disorder" OR "mood disorder" OR "memory disorder" OR "attention deficit hyperactivity disorder" OR "affective disorder" OR "stress-related disorder" |
| #4 | #1 AND #2 AND #3 |

11. medRxiv (search date: December 5, 2025 & June 18, 2026)

| #1 | (refugee OR displaced OR asylum seekers OR immigra* OR migrant) AND (artificial intelligence) AND (mental OR psychiatric) |
| --- | --- |

12. Google Scholar (search date: December 5, 2025)

| #1 | (refugee OR "asylum seeker" OR asylee OR "displaced person" OR "displaced population" OR "internally displaced" OR IDP* OR defector OR "humanitarian migrant" OR "forced migration" OR "forced displacement" OR "forcibly displaced" OR immigration OR immigrant OR migrant OR "forced migrant") AND  ("artificial intelligence" OR AI OR "large language model" OR LLM OR "natural language processing" OR NLP OR "machine learning" OR "deep learning" OR "supervised learning" OR "unsupervised learning" OR "neural network" OR "digital phenotyping" OR "predictive model" OR chatbot OR "rule-based" OR robotics OR "computer vision" OR "image generator") AND  (mental OR psychiatric OR psychosocial OR psychological OR cognitive OR depressed OR depression OR depressive OR anxiety OR PTSD OR trauma OR stress OR distress OR "posttraumatic stress" OR "post-traumatic stress" OR suicide OR suicidal OR suicidality OR bipolar OR sleep OR schizophrenia OR psychosis OR "obsessive compulsive disorder" OR "mood disorder" OR "memory disorder" OR "attention deficit hyperactivity disorder" OR "affective disorder" OR "stress-related disorder") |
| --- | --- |
| #4 | #1 AND #2 AND #3 |
