# Supplementary material for: AI-Based Approaches to Refugee Mental Health Care: Systematic Integrative Review
Source: J Med Internet Res. 2026 Aug 26;28:e101857. doi: 10.2196/101857 (PMC13559157; doi:10.2196/101857)
Supplement: Supplementary file 5 [file jmir_v28i1e101857_app5.docx]

**Multimedia Appendix 5.** Results of quality appraisal

1. Mixed Methods Appraisal Tool: Quantitative non-randomized

|  | S1 | S2 | 1.1 | 1.2 | 1.3 | 1.4 | 1.5 |
| --- | --- | --- | --- | --- | --- | --- | --- |
| Augsburger, 2017 [37] | Yes | Yes | No | Yes | Yes | Yes | Yes |
| Baird, 2022 [39] | Yes | Yes | Can’t tell | Yes | Yes | Yes | Yes |
| Qasrawi, 2022 [40] | Yes | Yes | Yes | Yes | Yes | Yes | Yes |
| Sahoo, 2024 [24] | Yes | Yes | Yes | Yes | Yes | Yes | Yes |
| Dell, 2024 [43] | Yes | Yes | Can’t tell | Yes | Yes | Yes | Yes |
| Saleh, 2024 [45] | Yes | Yes | Yes | Yes | Yes | Can’t tell | Yes |
| Figueiredo, 2025 [17] | Yes | Yes | Can’t tell | Yes | Yes | Yes | Yes |
| Keller, 2025 [50] | Yes | Yes | Can’t tell | Yes | Yes | Yes | Yes |
| Im, 2026 [52] | Yes | Yes | Yes | Yes | Yes | Yes | Yes |

S1: Are there clear research questions?

S2: Do the collected data allow to address the research question?

1.1. Are the participants representative of the target population?

1.2. Are measurements appropriate regarding both the outcome and intervention (or exposure)?

1.3. Are there complete outcome data?

1.4. Are the confounders accounted for in the design and analysis?

1.5. During the study period, is the intervention administered (or exposure occurred) as intended?

2. Mixed Methods Appraisal Tool: Quantitative descriptive

|  | S1 | S2 | 2.1 | 2.2 | 2.3 | 2.4 | 2.5 |
| --- | --- | --- | --- | --- | --- | --- | --- |
| Gellert, 2025 [51] | Yes | Yes | Yes | No | Can’t tell | Can’t tell | Yes |

S1: Are there clear research questions?

S2: Do the collected data allow to address the research question?

2.1. Is the sampling strategy relevant to address the research question?

2.2. Is the sample representative of the target population?

2.3. Are the measurements appropriate?

2.4. Is the risk of nonresponse bias low?

2.5. Is the statistical analysis appropriate to answer the research question?

3. Mixed Methods Appraisal Tool: Quantitative randomized controlled trials

|  | S1 | S2 | 3.1 | 3.2 | 3.3 | 3.4 | 3.5 |
| --- | --- | --- | --- | --- | --- | --- | --- |
| Demetry, 2026 [53] | Yes | Yes | Yes | Can’t tell | No | Yes | Yes |

S1: Are there clear research questions?

S2: Do the collected data allow to address the research question?

3.1. Is randomization appropriately performed?

3.2. Are the groups comparable at baseline?

3.3. Are there complete outcome data?

3.4. Are outcome assessors blinded to the intervention provided?

3.5. Did the participants adhere to the assigned intervention?

4. JBI Critical Appraisal Checklist for Text and Opinion Papers

|  | 1 | 2 | 3 | 4 | 5 | 6 |
| --- | --- | --- | --- | --- | --- | --- |
| Pozzi, 2025 [42] | Yes | Yes | Yes | Yes | Yes | Yes |

1. Is the source of the opinion clearly identified?

2. Does the source of opinion have standing in the field of expertise?

3. Are the interests of the relevant population the central focus of the opinion?

4. Is the stated position the result of an analytical process, and is there logic in the opinion expressed?

5. Is there reference to the extant literature?

6. Is any incongruence with the literature/sources logically defended?

**Reference**

[35] Hong QN, Fàbregues S, Bartlett G, Boardman F, Cargo M, Dagenais P, Gagnon MP, Griffiths F, Nicolau B, O’Cathain A, Rousseau MC. The Mixed Methods Appraisal Tool (MMAT) version 2018 for information professionals and researchers. Education for information. 2018 Nov;34(4):285-91.

[36] McArthur A, Klugárová J, Yan HU, Florescu S. Innovations in the systematic review of text and opinion. JBI Evidence Implementation. 2015 Sep 1;13(3):188-95.
